# Supplementary material for: Splice-Junction-Based Mapping of Alternative Isoforms in the Human Proteome
Source: Cell Rep. Author manuscript; Available in PMC 2020 Jan 15. (PMC6961840; doi:10.1016/j.celrep.2019.11.026)
Supplement: 2 [file NIHMS1546469-supplement-2.zip › DF1/PXD009737/TestisGluC/TestisGluC_18_RAD51D_QLINCLMLVSILEKPGLTEE.pdf]

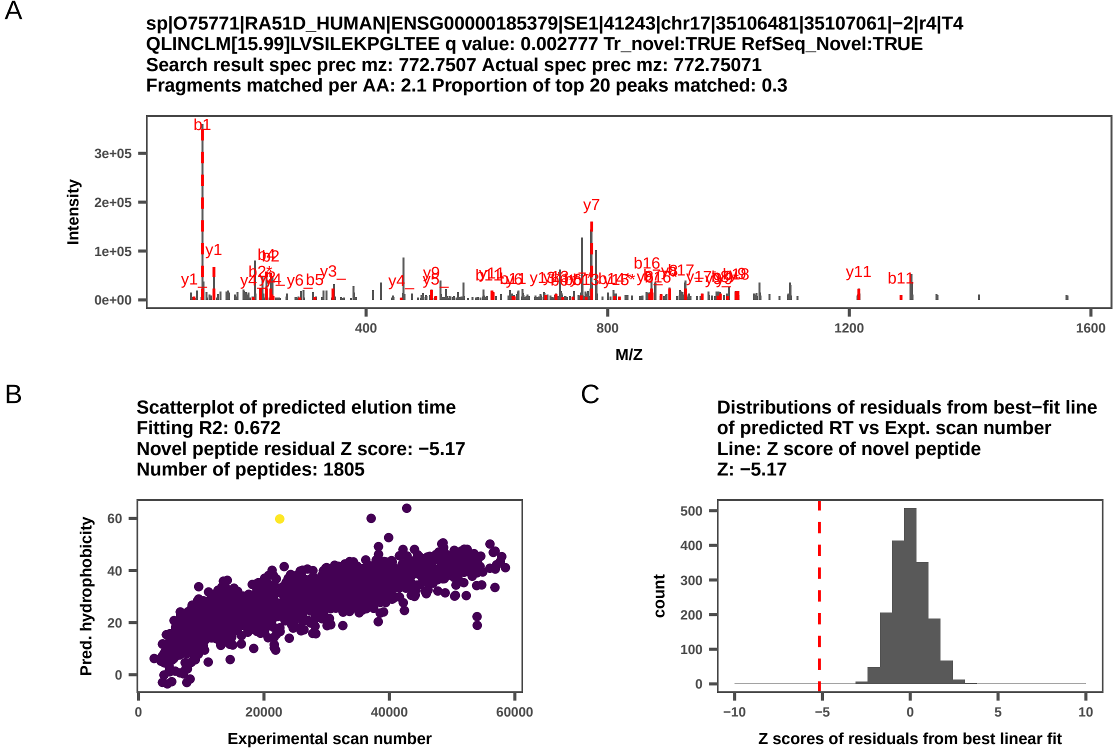
A
sp|O75771|RA51D_HUMAN|ENSG00000185379|SE1|41243|chr17|35106481|35107061|−2|r4|T4
QLINCLM[15.99]LVSILEKPGLTEE q value: 0.002777 Tr_novel:TRUE RefSeq_Novel:TRUE
Search result spec prec mz: 772.7507 Actual spec prec mz: 772.75071
Fragments matched per AA: 2.1 Proportion of top 20 peaks matched: 0.3
Intensity
3e+05
2e+05
1e+05
0e+00
M/Z
400
800
1200
1600
b1
y1
y7
y11
b11
b16
b17
B
Scatterplot of predicted elution time
Fitting R2: 0.672
Novel peptide residual Z score: −5.17
Number of peptides: 1805
Pred. hydrophobicity
60
40
20
0
Experimental scan number
0
20000
40000
60000
C
Distributions of residuals from best−fit line
of predicted RT vs Expt. scan number
Line: Z score of novel peptide
Z: −5.17
count
500
400
300
200
100
0
Z scores of residuals from best linear fit
−10
−5
0
5
10
